# Astragalus Injection for Hypertensive Renal Damage: A Systematic Review

**DOI:** 10.1155/2012/929025

**Published:** 2012-04-19

**Authors:** Tian Sun, Hao Xu, Fengqin Xu

**Affiliations:** ^1^Graduate School, Beijing University of Chinese Medicine, Beijing 100029, China; ^2^Cardiovascular Diseases Center, Xiyuan Hospital, China Academy of Chinese Medical Sciences, Beijing 100091, China; ^3^Cadres Department, Xiyuan Hospital, China Academy of Chinese Medical Sciences, Beijing 100091, China

## Abstract

*Objective*. To evaluate the effectiveness of astragalus injection (a traditional Chinese patent medicine) for patients with renal damage induced by hypertension according to the available evidence. *Methods*. We searched MEDLINE, China National Knowledge Infrastructure (CNKI), Chinese VIP Information, China Biology Medicine (CBM), and Chinese Medical Citation Index (CMCI), and the date of search starts from the first of database to August 2011. No language restriction was applied. We included randomized controlled trials testing astragalus injection against placebo or astragalus injection plus antihypertensive drugs against antihypertensive drugs. Study selection, data extraction, quality assessment, and data analyses were conducted according to the Cochrane review standards. *Results*. 5 randomized trials (involving 429 patients) were included and the methodological quality was evaluated as generally low. The pooled results showed that astragalus injection was more effective in lowering *β*_2_-microglobulin (*β*_2_-MG), microalbuminuria (mAlb) compared with placebo, and it was also superior to prostaglandin in lowering blood urea nitrogen (BUN), creatinine clearance rate (Ccr). There were no adverse effects reported in the trials from astragalus injection. *Conclusions*. Astragalus injection showed protective effects in hypertensive renal damage patients, although available studies are not adequate to draw a definite conclusion due to low quality of included trials. More rigorous clinical trials with high quality are warranted to give high level of evidence.

## 1. Introduction

Hypertensive renal damage has been defined as being characterized by the changes in renal structure and function which was caused by hypertension. Renal damage is one of three hypertensive complications. Hypertension could cause renal damage in early stage, and the renal damage often happens insidiously and persists many years without any typical clinical symptoms. In the past ten years, the incidence of end-stage renal disease (ESRD) was rising at an annual rate of 9% and 28% was caused by hypertension [1]. In recent years, the incidence of ESRD caused by hypertension was also increased in China [2].

At present, antihypertensive drugs have been shown to be effective in lowering blood pressure and thus reducing morbidity and mortality of cardiovascular diseases. Angiotensin-converting enzyme inhibitors (ACEI) or angiotensin II receptor blocker (ARB) could also exert kidney protective effect by dilating efferent arterioles more than afferent arteriole, decreasing urinary albumin, and inhibiting the glomerulosclerosis. However, the treatment of hypertensive renal damage still needs to be further improved even on the basis of ACEI or ARB. Astragalus injection is a preparation of an extract of Radix Astragali. The major components are astragalosides [3], and the other pharmacological ingredients include polysaccharides, flavones, and aminoacids. Modern pharmacological research has indicated that astragalus injection could enhance myocardial contractility, improve circulation, protect myocardial cells and regulate immune function [4, 5]. Recent reviews [6–8] further indicated the potential benefit of astragalus injection in the treatment of hypertensive renal damage. The following systematic review aims to test whether astragalus injection is effective and safe in treating hypertensive renal damage.

## 2. Methods

### 2.1. Database and Search Strategies

We searched MEDLINE, China National Knowledge Infrastructure (CNKI), Chinese VIP Information, China Biology Medicine (CBM), and Chinese Medical Citation Index (CMCI). The date of search was from the first of database start to August 2011. No language restrictions were applied. We used the terms “hypertensive renal damage”, “hypertensive renal injury”, “astragalus injection”, and “Huangqi injection”. Various combinations of the terms were used, depending on the database searched.

### 2.2. Inclusion Criteria

(1) Randomized controlled trials (RCT); (2) male or female patients, of any age or ethnic origin, who had hypertensive renal damage. Hypertensive renal damage was diagnosed on the basis of: (i) a history of essential hypertension, (ii) persistent proteinuria, (iii) hypertensive retinopathy, (iv) primary renal diseases or other secondary renal disease was excluded; (3) the intervention measure was astragalus injection, astragalus injection plus placebo, or astragalus injection plus antihypertensive drugs; (4) all trials had to report clinically relevant outcome measures of hypertensive renal damage; (5) the treatment should be at least two weeks. Outcome measures include results of blood pressure, renal function, clinical comprehensive effect, and Traditional Chinese medicine (TCM) syndrome differentiation. Duplicated publications reporting the same groups of participants were excluded.

### 2.3. Data Extraction and Quality Assessment

Two reviewers (T. Sun, H. Xu) extracted data independently. We assessed the methodological quality of all included trials by using the table of risk of bias provided by RevMan 5.1.0. The scale consists of seven items pertaining to description of random sequence generation, allocation concealment, blinding of participants and personnel, blinding of outcome assessment, incomplete outcome data, selective reporting, and other bias.

### 2.4. Data Synthesis

We used RevMan 5.1.0 provided by Cochrane Collaboration to analyse the data. Dichotomous data were expressed as relative risk (RR) and continuous outcomes as weighted mean difference (WMD), both with 95% confidence intervals (CI). Heterogeneity was assessed using the *I* ^2^ test with the significance level set at *I* ^2^ over 50% or *P* < 0.1. In the absence of significant heterogeneity, we pooled data using a fixed-effect model (*I* ^2^ < 50%), otherwise we using random effects model (*I* ^2^ > 50%) [9].

## 3. Results

### 3.1. Description of Included Trials

**Figure 1:**
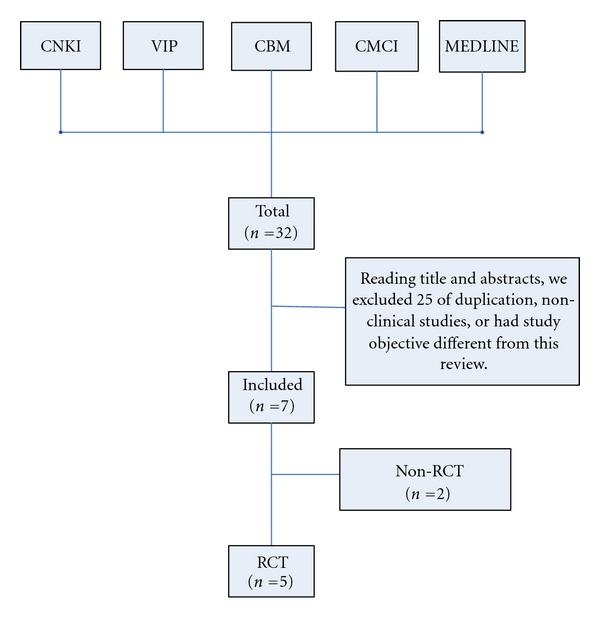
Flow diagram of the article selection for this study.

Our search identified 32 references. We excluded 27 of these articles. Flow diagram of the article selection for this study is shown in Figure 1.

The search yielded 5 eligible trials, which were all conducted and published in China. A total of 429 participants with renal damage induced by hypertension were included in the 5 trials. The proportion of male participants was 66.8%. All the trials included inpatients, and the average size of the trials was 86 patients (ranging from 48 to 127 participants). Five trials enrolled patients with renal damage induced with hypertension. The diagnostic criteria of trials were based on the guidelines for prevention and treatment of hypertension in China [4, 5], clinical manifestations, and laboratory tests [3, 6, 7]. Three trials were astragalus injection combined with antihypertensive drugs against antihypertensive drugs, one trial was astragalus injection against placebo, and one trial was astragalus injection against prostaglandin. No trial reported outcomes of the incidence of complications, health economic costs, quality of life, or adverse effects. The outcomes that were reported included twenty-four hours urinary protein content, microalbuminuria (mAlb), *β*_2_-microglobulin (*β*_2_-MG), blood urea nitrogen (BUN), serum creatinine (Scr), and creatinine clearance rate (Ccr). Characteristics of included studies were shown in Table 1.

### 3.2. Methodological Quality of Included Trials

**Figure 2:**
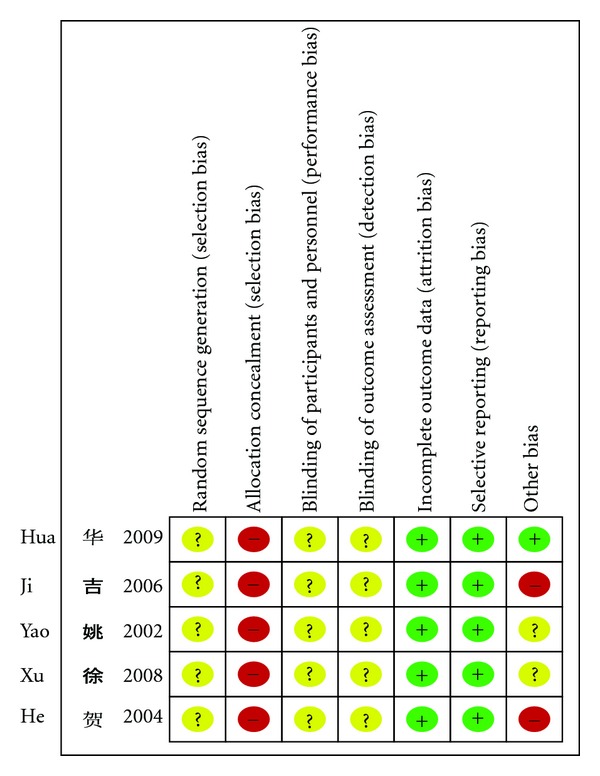
Risk of bias summary.

The methodological quality of all the five trials was very low (Figure 2): none of trials reported sample calculation, the sample size of trials was small. These trials provided limited information on allocation concealment and blinding, and they were all lack of description of the allocation sequence generation. All the trials did not mention followup. We contacted the author for further information but regrettably no information has been provided to date.

### 3.3. Effect of Interventions

Three trials [3, 5, 7] gave biochemical indices to analyse the effective of astragalus injection. One trial [4] only gave the number of patients who had symptomatic improvement, and one trial [6] gave both biochemical indices and the number of patients who had symptomatic improvement. All were showed in Tables 2 and 3.

#### 3.3.1. The Analysis of Improvement of Renal Damage Indices

It was not possible to pool the data on renal damage indicators, since the results describing varied indicators to prove the curative effect of astragalus injection.

In the Ji trial [10], the experimental group used astragalus injection (*n* = 54), while glucose injection was administered in the control group. Astragalus injection showed significant effect on indicators of *β*_2_-MG (MD −15.14, 95%CI −21.61 to −8.67) and mAlb (MD −28.41, 95%CI 47.67 to −9.15).

Xu trial [11] used astragalus injection combined with Telmisartan and Plendil in the experimental group (*n* = 26), while Telmisartan and Plendil were administered in the control group. Only indicators of pulse pressure (MD −7.00, 95%CI −11.56 to −2.44), systolic blood pressure (SBP) (MD −21.70, 95%CI −31.24, −12.16), and twenty-four hours urinary protein content (MD −0.05, 95%CI −0.07, −0.04) showed significant differences.

In He trial [12], the experimental group used astragalus injection (*n* = 50), and prostaglandin was used in the control group. Astragalus injection showed significant effect on indicator of BUN (MD −7.39, 95%CI −9.83, −4.95) and Ccr (MD 6.84, 95%CI 4.57, 9.11).

In Yao trial [13], there was no significant difference between astragalus injection plus Lotensin and Plendil group and Lotensin and Plendil group, according to indicator of twenty-four hours urinary protein content.

#### 3.3.2. Symptoms and Signs

There were only two trials who reported the improvement on symptoms and signs (Table 3). However, they were all for comprehensive therapeutic effect. We cannot obtain the number of patients with individual symptoms and the data of individual symptoms improvement after treatment. So we cannot get the analysis of comparison between groups.

### 3.4. Final Indicator at Endpoint

None of the trial reported the mortality rate or the incidence of complication.

### 3.5. Sensitivity Analysis, Subgroup Analysis, and Publication Bias

The number of trials was too small to conduct any sufficient additional analysis of sensitivity, subgroup, and publication bias.

### 3.6. Adverse Reaction

None of the trial reported the observation of side effects.

## 4. Discussion

Our systematic review suggested that astragalus injection may be effective on laboratory indices of renal damage (*β*_2_-MG, mAlb, pulse pressure, SBP, BUN, Ccr) or improvement of symptoms and signs. However, according to potential publication bias and low-quality trials, available data are not adequate to draw a definite conclusion of astragalus injection in treating renal damage induced by hypertension. More specifically, the positive findings should be interpreted conservatively due to the following facts.

The five trials included in this paper had risk of bias in terms of design, reporting, and methodology. They provided only limited descriptions of study design, allocation concealment, and baseline data. All the five RCTs prohibited us from performing meaningful sensitivity analysis. The included trials were heterogeneous in the populations (adults, elderly people) and the reported outcomes. All the included trials were not multicenter, large scale RCTs.

The primary goal of treatment for renal damage induced by hypertension is to prevent death or progression to complications. The outcomes from all the included trials are mainly laboratory indices and symptom improvement. There is a lack of data from all the trials on clinically relevant outcomes such as the mortality, incidence of complications, and quality of life.

Nevertheless, astragalus injection is administered for treating renal damage induced by hypertension in China. We have identified more than 30 randomized trials on this topic until now. However, most of them are not eligible for the review due to inadequate design, conducting, and reporting of the trials. Chinese researchers must be aware of the need to design and use appropriate statistical methods in future RCTs of astragalus injection and to measure clinical outcomes rather than physiological (surrogate) outcomes.

All the five trials did not report that adverse events. A conclusion about the safety of astragalus injection cannot be made. In China, it is widely believed that it is safe to use herbal medicines for various conditions. All the trials did not report that adverse events may reflect current situation. However, the safety of herbal medicines needs to be monitored carefully and reported appropriately in the future clinical trials. In fact, we found that some reports [14–16] indicated that astragalus injection had adverse outcomes.

Although we conducted comprehensive searches, we only identified and included trials published in Chinese. Most of the trials are small sample with positive findings. We tried to avoid language bias and location bias, but we cannot exclude potential publication bias. We have conducted extensive searches for unpublished material, but at the same time we cannot neglect the fact that trials with negative findings remain unpublished.

Based on this systematic review, the effectiveness and safety of astragalus injection in patients with hypertensive renal damage is uncertain. The evidence is inconclusive due to poorly designed and low-quality trials. There is a need for additional RCTs that emphasize not only good clinical design but also more elaborated description of the intervention and clinically relevant outcomes including the mortality, incidence of complications, and quality of life.

## Figures and Tables

**Table 1:** Characteristics of Included Studies.

Study ID	Gender male/female	Base-line information	Average age (years)	Interventions	Control	Duration of treatment (days)	Outcome measures
Ji and Yin 2006 [10]	59/35	Age, sex, condition	45.2	Astragalus injection (250 mL i.d, qd)	5% glucose injection	30	*β*_2_-MG, mAlb
Hua et al. 2009 [17]	84/43	Age, sex, blood pressure, duration	44.3	Astragalus injection (250 mL i.d, qd) calcium channel blockers (CCB), ACEI/ARB, thiazine diuretics	CCB,ACEI/ARB, thiazine diuretics	20	Twenty-four hours urinary protein content, blood pressure, urinalysis, renal function
Xu et al. 2008 [11]	28/20	Age, sex, blood pressure	63.6 ± 14.1	Astragalus injection (250 mL i.d, qd) Telmisartan, Plendil	Telmisartan, Plendil	21	Twenty-four hours urinary protein content, blood pressure, mAlb, serum potassium, pulse pressure, estimated glomerular filtration rate (eGFR)
He 2004 [12]	78/18	Age, sex	74.2	Astragalus injection (500 mL i.d, qd)	Prostaglandin (PGE1)	15	BUN, Scr, Ccr
Yao et al. 2002 [13]	38/26	Age, sex, blood pressure	64.7 ± 13.7	Astragalus injection (250 mL i.d, qd) Lotensin, Plendil	Lotensin, Plendil	21	Twenty-four hours urinary protein content

**Table 2:** The Analysis of Improvement of renal damage indices.

Renal damage indices and comparison between the groups	No. of studies	WMD [95% CI]	*P* value
**β**_2_-MG			
Astragalus versus glucose injection [10]	1	−15.14 [−21.61, −8.67]	*P* < 0.00001
mAlb			
Astragalus versus glucose injection [10]	1	−28.4 [−47.67, −9.15]	*P* = 0.004
Astragalus plus Telmisartan, Plendil versus Telmisartan, and Plendil [11]	1	−4.20 [−7.47, −0.93]	*P* = 0.01
eGFR			
Astragalus plus Telmisartan, Plendil versus Telmisartan, and Plendil [11]	1	4.10 [−2.38, 10.58]	*P* = 0.21
pulse pressure			
Astragalus plus Telmisartan, Plendil versus Telmisartan, and Plendil [11]	1	−7.00 [−11.56, −2.44]	*P* = 0.003
SBP			
Astragalus plus Telmisartan, Plendil versus Telmisartan, and Plendil [11]	1	−21.70 [−31.24, −12.16]	*P* < 0.00001
DBP			
Astragalus plus Telmisartan, Plendil versus Telmisartan, and Plendil [11]	1	−4.20 [−11.02, 2.62]	*P* = 0.23
serum potassium			
Astragalus plus Telmisartan, Plendil versus Telmisartan, and Plendil [11]	1	−0.08[−0.28, 0.12]	*P* = 0.44
twenty-four hours urinary protein content			
Astragalus plus Telmisartan, Plendil versus Telmisartan, and Plendil [11]	1	−0.05 [−0.07, −0.04]	*P* < 0.00001
Astragalus plus Lotensin, Plendil versus Lotensin, and Plendil [13]	1	−0.21 [−0.50, 0.08]	*P* = 0.15
BUN			
Astragalus versus prostaglandin [12]	1	−7.39 [−9.83, −4.95]	*P* < 0.00001
Scr			
Astragalus versus prostaglandin [12]	1	−3.37 [46.05, 39.31]	*P* = 0.88
Ccr			
Astragalus versus prostaglandin [12]	1	6.84 [4.57, 9.11]	*P* < 0.00001

**Table 3:** The analysis of comprehensive therapeutic effect.

Symptom and sign	No. of studies	Intervention *(n/N) *	Control *(n/N) *	RR [95%CI]	*P* value
Astragalus plus thiazine diuretics versus thiazine diuretics	1	10/64	32/64	0.18 [0.08, 0.41]	*P* < 0.00001
Astragalus versus prostaglandin	1	2/50	13/46	0.11 [0.02, 0.50]	*P* = 0.005
